# Building a SHAP interpretable prediction model for stroke-associated pneumonia [stroke-associated pneumonia (SAP)] in patients with aneurysmal subarachnoid hemorrhage (aSAH)

**DOI:** 10.3389/fneur.2026.1666960

**Published:** 2026-04-17

**Authors:** Yuqi Zhang, Mingjian Lin, Yun-Xiang Zhou, Xiaoyong Lin, Yingcong Wei, Bo Li, Jintang Li, Honghai Luo, Yifan Deng, Zhongzong Qin, Gang Zhu

**Affiliations:** 1 Guangdong Medical University, Zhanjiang, Guangdong, China; 2 Department of Neurosurgery, Huizhou Central People’s Hospital, Huizhou, Guangdong, China; 3 Department of Neurosurgery, Gaozhou People’s Hospital, Maoming, Guangdong, China; 4 Department of Neurosurgery, The First Affiliated Hospital of Shaoyang University, Shaoyang, China

**Keywords:** aneurysmal subarachnoid hemorrhage, machine learning, nomogram, prediction model, SHAP interpretable model, stroke-associated pneumonia

## Abstract

**Background:**

Aneurysmal subarachnoid hemorrhage (aSAH) is a neurological emergency characterized by intracranial aneurysm rupture, leading to blood influx into the subarachnoid space and imposing high mortality and disability rates. The aim of this study was combining the epidemiological characteristics of aSAH and the high incidence of stroke-associated pneumonia (SAP), we aimed to develop a SHAP-explainable prediction model that may provide clinical reference value, providing a new approach to reduce the burden of complications. We take the random forest (RF) model combined with SHAP interpretation as the primary predictive model, and the logistic regression-based nomogram as a supplementary transparent tool for clinical bedside application.

**Methods:**

A retrospective analysis was conducted in aSAH patients at Huizhou Central People’s Hospital. The patients were randomly split into training and validation sets for methodological purposes to generate and validate a SHAP interpretable machine learning model.

**Results:**

A total of 375 patients were initially enrolled, with 85 excluded due to exclusion criteria, leaving 290 patients for retrospective analysis. Univariate logistic regression, LASSO regression, and Boruta algorithm were used for multivariable feature selection and the common factors across the three methods were lactate dehydrogenase (LDH, SIRI, BMI, Age, AST, D-Dimer, and Hunt–Hess score). We established a nomogram model with good predictive performance, low error rate, and significant clinical benefits. The RF model showed stable performance and good efficacy in both training and validation sets. Based on the RF model, SHAP analysis was conducted to evaluate risk factor importance and individual impacts. The RF model with SHAP interpretation was identified as the primary predictive model, while the nomogram served as a supplementary transparent tool.

**Conclusion:**

This study identifies postoperative stroke-associated pneumonia [stroke-associated pneumonia (SAP)] within 14 days after endovascular embolization BMI, Age, SIRI, Hunt–Hess score, D-Dimer, AST, and lactate dehydrogenase LDH as key predictors of postoperative stroke-associated pneumonia (SAP) in aSAH, and demonstrates the efficient performance of the RF random forest model (with SHAP interpretation) in prediction.

## 1 Introduction

Aneurysmal subarachnoid hemorrhage (aSAH) is a neurological emergency characterized by intracranial aneurysm rupture, leading to blood influx into the subarachnoid space and imposing high mortality and disability rates (1, 2). Although relatively rare among all stroke types, aSAH imposes a huge burden on healthcare systems and individual health due to its catastrophic consequences (2, 3). The in-hospital mortality rate of aSAH reaches up to 20%, highlighting the urgency of emergency management (4). Epidemiological features of aSAH show an increased incidence in specific populations (e.g., late pregnancy) and identify it as the main cause of non-traumatic subarachnoid hemorrhage (5). A national inpatient sample study (2000–2019) showed that the prognosis of aSAH patients is influenced by factors such as gender, race, and ethnicity, further reflecting the complexity of its epidemiology (6). Additionally, during the COVID-19 pandemic, the burden of strokes like subarachnoid hemorrhage has trended upward, emphasizing the need for public health attention (7). Recent data show that aSAH accounts for 5–10% of all strokes but contributes to 20% of stroke-related mortality, highlighting its disproportionate impact.

Stroke-associated pneumonia (stroke-associated pneumonia (SAP)), defined as lung infections related to stroke (e.g., postoperative pneumonia or hospital-acquired pneumonia), is a common complication after aSAH and a major driver of poor prognosis (8–10). In this study, stroke-associated pneumonia (SAP) refers to postoperative lung infection occurring within 14 days after aSAH endovascular embolization, consistent with the diagnostic criteria in the Methods section. Clinically, stroke-associated pneumonia (SAP) has a high incidence in aSAH patients, with variations across populations. For example, a retrospective analysis of 308 aSAH surgical patients showed that pneumonia was the main factor leading to poor prognosis; in another cohort of 253 aSAH patients, the incidence of hospital-acquired pneumonia (HAP) was 25.3% (64/253) (11). In broader stroke populations, the stroke-associated pneumonia (SAP) incidence is approximately 26.55% (73/275) in acute ischemic stroke patients (12) and up to one-third (33%) in intracerebral hemorrhage patients (13). This high incidence is associated with severe consequences of stroke-associated pneumonia (SAP), including prolonged hospital stays, increased medical costs, and doubled mortality risk, exacerbating the overall burden on aSAH patients (14, 15). For instance, one study indicated that stroke-associated pneumonia (SAP) is a key factor in mortality and disability after aSAH, emphasizing the importance of early identification and active intervention (16).

However, existing stroke-associated pneumonia (SAP) prediction models (e.g., traditional clinical scores or simple logistic regression models) often lack interpretability and accuracy, limiting their clinical application (17, 18). Many models rely solely on baseline data, ignoring dynamic factors such as imaging (e.g., CT scans) and inflammatory markers (19). Existing stroke-associated pneumonia (SAP) prediction tools (e.g., A^2^DS^2^ score) are primarily designed for general stroke populations, and few aSAH-specific stroke-associated pneumonia (SAP) prediction models exist. Although existing tools (e.g., Bayesian networks or machine learning models) improve prediction performance, they lack interpretability, failing to clarify the mechanisms of key risk factors (e.g., inflammatory indices or respiratory function parameters) (20). Therefore, constructing a SHAP (SHapley Additive exPlanations)-based explainable prediction model is particularly important (21, 22). SHAP analysis can enhance model transparency by identifying and explaining key predictive variables (e.g., postoperative neutrophil-to-lymphocyte ratio [NLR] or mechanical ventilation status), providing insights for clinicians to develop potential individualized intervention strategies (e.g., early antibiotic therapy or respiratory support) (23). In summary, combining the epidemiological characteristics of aSAH and the high incidence of stroke-associated pneumonia (SAP), developing a SHAP-explainable model has significant clinical value, providing a new approach to reduce the burden of complications.

## 2 Materials and methods

### 2.1 Patient selection

This retrospective study analyzed the clinical data of 290 patients who underwent aneurysm embolization at Huizhou Central People’s Hospital from January 2019 to December 2023.

*Inclusion criteria*: (1) Diagnosed with aneurysmal subarachnoid hemorrhage by computed tomography (CT), CT angiography, or digital subtraction angiography. (2) Met surgical indications and could tolerate surgery. (3) Signed informed consent for surgery. (4) Received endovascular embolization after admission. (5) Aged >18 years.

All patients requiring mechanical ventilation in this study followed a unified management protocol: assist-control ventilation (ACV) mode was adopted, with tidal volume set to 6–8 mL/kg of ideal body weight, PEEP maintained at 5–8 cmH₂O, and FiO_2_ adjusted according to arterial blood gas to maintain SpO_2_>94%. Daily assessment of weaning indicators was performed, and spontaneous breathing trials (SBT) were initiated for eligible patients. This protocol was developed with reference to the Chinese Expert Consensus on Respiratory Support for Neurosurgical Critical Care Patients to ensure consistency in ventilator management within the center. Adherence to the ventilator protocol was 92.3%. No major deviations in PEEP/FiO_2_ targets; 3 cases of delayed SBT due to neurological deterioration. Sedation was standardized with propofol (RASS target = −1 to 0) and analgesia with fentanyl. Airway care included daily chlorhexidine oral hygiene, 30° head-of-bed elevation, and early mobilization within 48 h (if neurologically stable). These measures were standardized across all patients, so no adjustment was needed as covariates.

*Exclusion criteria*: (1) Patients in a dying state, ineligible for surgery, or with no surgical benefit, or patients/relatives who signed to abandon treatment. (2) Diagnosed with community-acquired pneumonia or pre-admission lung infection. (3) Pseudoaneurysm. (4) Comorbid severe cardiovascular diseases, hematological diseases, or other serious illnesses. (5) Incomplete medical records. (6) Aged <18 years. (7) Non-aneurysmal subarachnoid hemorrhage (e.g., arteriovenous malformation, perimesencephalic benign hemorrhage, traumatic intracerebral hemorrhage).

The sample size power analysis was based on the following assumptions: ① *α* = 0.05 (two-sided), *β* = 0.2 (power 80%); ② The expected OR of major predictors (e.g., Age, Hunt–Hess score) was 1.5; ③ The incidence of stroke-associated pneumonia (SAP) was set to 25.9% (75/290) based on previous data from our center. Calculations using PASS 15.0 software showed that a minimum of 210 samples were required to detect predictors with OR > 1.5. This study actually included 290 cases (203 in the training set and 87 in the validation set), including 75 cases in the stroke-associated pneumonia (SAP) group and 215 cases in the non-stroke-associated pneumonia (SAP) group, with a power of 89.3%, which meets the requirements of statistical testing. Although the sample size of the validation set (87 cases) is relatively small, Bootstrap resampling (1,000 times) verified that the AUC fluctuation range of the model was 0.72–0.78, indicating stable performance.

As a retrospective study, the ethics committee approved the waiver of informed consent in accordance with national laws and institutional regulations. Patient personal identification information was anonymized in this study.

### 2.2 Data collection and definition

We systematically collected and analyzed comprehensive clinical data of 290 patients who underwent coil embolization for ruptured intracranial aneurysms (RIAs) from 2019 to 2023 via the electronic medical record retrieval system of Huizhou Central People’s Hospital. The data included basic information (age, gender) and medical history characteristics (hypertension, diabetes, smoking history, alcohol consumption history). Additionally, the Hunt–Hess grading system and Glasgow Coma Scale (GCS) were used to assess the severity of the patients’ condition on admission, along with admission systolic and diastolic blood pressure data. This comprehensive data collection provided valuable information for exploring the clinical characteristics and influencing factors of RIA embolization patients.

All laboratory data included BMI, hemoglobin (Hb), red blood cell count (RBC), hematocrit (HCT), mean corpuscular volume (MCV), mean corpuscular hemoglobin (MCH), mean corpuscular hemoglobin concentration (MCHC), red cell distribution width (RDW), white blood cell count (WBC), neutrophils (Neut), lymphocytes (Lymph), monocytes (Mono), eosinophils (Eo), basophils (Baso), platelet count (Plt), plateletcrit (Pct), mean platelet volume (Mpv), platelet distribution width (Pdw), prealbumin (Pab), sodium (Na), chloride (Cl), potassium (K), calcium (Ca), urea, creatinine (Crea), total protein (Tp), albumin (Alb), albumin/globulin ratio (AGR), total bilirubin (TBIL), direct bilirubin (DBIL), alanine aminotransferase (ALT), aspartate aminotransferase (AST), alkaline phosphatase (ALP), gamma-glutamyl transferase (GGT), lactate dehydrogenase lactate dehydrogenase (LDH), adenosine deaminase (ADA), fibrinogen (FIB), D-Dimer, hypertension history, diabetes history, systolic blood pressure (SBP), diastolic blood pressure (DBP), GCS score, Hunt–Hess Score (Hunt–Hess score), neutrophil-to-lymphocyte ratio (NLR), systemic immune-inflammation index (SII), prognostic nutritional index (PNI), systemic inflammation response index (SIRI), and platelet-to-lymphocyte ratio (PLR).

### 2.3 Outcome

In this study, stroke-associated pneumonia (SAP) refers to hospital-acquired pneumonia occurring within 14 days after aSAH endovascular embolization, excluding ventilator-associated pneumonia (VAP) unrelated to surgical intervention and community-acquired pneumonia before admission.

stroke-associated pneumonia (SAP) was independently adjudicated by two senior attending physicians who were blinded to the patient’s clinical data and other study results; the Kappa consistency test was used to evaluate the adjudication result, with Kappa = 0.89 (95% CI:0.81–0.97), indicating excellent inter-rater consistency.

The diagnostic criteria are classified into mandatory and optional criteria: (1) Mandatory criteria: new pneumonia ≥48 h after admission (no community-acquired infection), no other clear infection sources, and chest X-ray/CT showing new/progressive infiltration/consolidation; (2) Optional criteria: at least 2 of the following clinical manifestations (one must be respiratory-related): new/worsening cough/expectoration, pulmonary rales/consolidation signs, body temperature >38 °C/<36 °C, WBC > 10 × 10^9^/L/ < 4 × 10^9^/L, decreased oxygenation.

1. Basic conditions: Patients must meet all of the following: new pneumonia ≥48 h after admission (excluding community-acquired infection); no other clear infection sources (e.g., urinary tract infection, abdominal infection leading to lung manifestations).
2. Clinical manifestations: At least 2 clinical criteria (1 must be respiratory symptoms or signs): Respiratory symptoms: New or worsening cough, expectoration, purulent sputum; Signs: Pulmonary rales, consolidation signs; Systemic inflammatory response: Body temperature >38 °C or <36 °C; white blood cell count >10 × 10^9^/L or <4 × 10^9^/L; Deterioration of oxygenation: Requirement for increased oxygen concentration or mechanical ventilation support (e.g., decreased PaO₂/FiO_2_ ratio).
3. Imaging evidence: Chest X-ray or CT showing new or progressive infiltration, consolidation, or cavitation.

*Etiological detection methods*: Sputum culture (qualified sputum specimen: squamous epithelial cells <10/HPF); bronchoalveolar lavage (BAL) or protected specimen brush (PSB) (for severe or mechanically ventilated patients); blood culture (when bacteremia is suspected); urinary antigen detection (e.g., Legionella, *Streptococcus pneumoniae*); molecular biology testing (e.g., PCR for drug-resistant genes). Stroke-associated pneumonia (SAP) was independently evaluated and diagnosed by two senior attending physicians.

### 2.4 Statistical analysis

Fixed random seeds are used for all random processes in the following statistical analysis to ensure the reproducibility of the study results.

#### 2.4.1 Multiple imputation

We used multiple imputation to handle missing data. The raw dataset was first randomly split into training (70%, *n* = 203) and validation (30%, *n* = 87) sets using a fixed random seed (seed = 123) – a ratio consistent with common prediction model development practices. This order avoids information leakage from the validation set to the training set, which is consistent with the standard practice of prediction model development. Multiple imputation involves generating random values to fill missing data via statistical models, producing multiple complete datasets. Multiple imputation was performed only on the training set (*m* = 5 imputed datasets), and the same imputation model was applied to the validation set to avoid information leakage. The imputed original dataset was randomly divided into a training set (203 samples) and a validation set (87 samples) at a 7:3 ratio, ensuring consistent data distribution and equal allocation probability. A sensitivity analysis comparing model performance between training-set-only imputation and full-dataset imputation showed no significant difference (AUC difference < 0.02), confirming robustness. Multiple imputation was performed using MICE (predictive mean matching, PMM) with *m* = 5 imputed datasets. The imputation model included all predictors and the outcome. Convergence was confirmed via trace plots (no evidence of non-convergence). A complete-case sensitivity analysis showed AUC = 0.73 (consistent with imputed model AUC = 0.76), confirming robustness.

#### 2.4.2 Feature selection

This study prespecifies that multivariate logistic regression is used to identify linear independent predictive factors, while the ML model will incorporate all seven common factors screened by the three methods to capture non-linear interactions and multi-dimensional risk signals of stroke-associated pneumonia (SAP), which is a pre-designed research strategy rather than a post-hoc adjustment. The multi-step feature selection includes three sequential steps: (1) Exploratory univariate logistic regression (*p* < 0.05 as preliminary threshold); (2) Multivariable feature selection via LASSO regression (*λ* = Lambda.min for optimal accuracy) and Boruta algorithm (retaining ‘confirmed important’ features); (3) Selection of common factors from the above three methods combined with clinical plausibility to determine final predictors. Univariate logistic regression analysis: Used for multi-step feature selection of training set data. Data were collected and univariate logistic regression was performed for each independent variable to calculate regression coefficients, odds ratios (OR), 95% confidence intervals (CI), and *p*-values. A *p*-value < 0.05 was considered statistically significant.

*LASSO regression*: a multivariate linear regression model with L1 regularization, used for feature selection and reducing model complexity. Unlike multi-step feature selection, LASSO considers the correlation between variables and penalizes the absolute values of regression coefficients, forcing irrelevant variables to have coefficients of 0. Process: The process involves data preprocessing (handling missing values, outliers, and normalizing features), dividing the data into training and test sets, fitting the multivariate LASSO model on the training subset (inner loop of NCV), determining the optimal regularization parameter *λ* via cross-validation (using Lambda.min for optimal accuracy) to select features with non-zero coefficients and retain these features for subsequent model construction, fitting the LASSO model with the training set, and finally evaluating the model performance using the test set. Boruta algorithm: A random forest-based feature selection algorithm. Process: Data preparation (factorizing categorical variables); executing the Boruta algorithm to assess feature importance; extracting important features (classified as confirmed important, confirmed unimportant, or uncertain); visualizing feature importance.

To explore the association relationship between key predictors (e.g., SIRI, Hunt–Hess score) and stroke-associated pneumonia (SAP), this study supplemented the analysis using the target trial emulation framework^1^: ① “High SIRI (>1.5)” was defined as the exposed group, and “low SIRI (≤1.5)” as the control group; the SIRI threshold (>1.5) was prespecified in the study design stage, based on the retrospective pre-experiment data of 100 aSAH patients in our center (2019 before) and relevant clinical studies (37892156), which is not a post-hoc determined threshold. ② Propensity score matching (PSM) was used to balance confounding factors (such as age, BMI, and Hunt–Hess score) between the two groups (matching ratio 1:1, caliper 0.2); ③ Intent-to-treat (ITT) analysis was performed to compare the difference in stroke-associated pneumonia (SAP) incidence between the exposed group and the control group and the risk ratio (RR) and 95% confidence interval (CI) were calculated to verify the causal effect of SIRI on stroke-associated pneumonia (SAP).

The intersection of results from the three multi-step feature selection methods was considered the most predictive factors, which were then incorporated into a multivariate logistic regression model to identify true influencing factors.

#### 2.4.3 Nomogram

*ROC curves for training and validation sets*: Data were divided into training and validation sets at a 7:3 ratio. A logistic regression model was selected, trained using the training set, and ROC curves were plotted by calculating prediction probabilities for the validation set.

*Calibration curves for training and validation sets*: Patients were sorted by predicted probability, divided into equal parts, and a scatter plot was drawn with the mean predicted probability as the x-axis and the actual event occurrence rate as the y-axis. An ideal calibration curve should be a 45° line.

*DCA curves for training and validation sets*: The training set DCA curve evaluates model performance on training data, while the validation set DCA curve assesses generalization ability. Similar curves indicate good model generalization.

#### 2.4.4 Machine learning algorithms and SHAP (shapley additive explanations) interpretation

This study prespecifies the RF model as the primary analytic model for stroke-associated pneumonia (SAP) prediction, and SHAP analysis is exclusively applied to the RF model for interpretability; the nomogram based on multivariate logistic regression is a supplementary tool, which is designed to convert the complex ML model into a visual scoring system for convenient clinical use.

Machine learning algorithms are mathematical models and statistical algorithms that enable computer systems to learn from data and improve performance, featuring pattern learning, prediction/classification, and model optimization. SHAP values, based on the game theory concept of Shapley values, measure the contribution of each feature to model predictions. Potential predictive factors were incorporated into multiple machine learning algorithms (RF, XGB, KNN, DT, LR, SVM, LGBM) for ROC comparison. The prespecified primary model is the RF model for prediction, with SHAP analysis applied exclusively to the RF model for interpretability. The nomogram (based on logistic regression) serves as a comparative reference. All models were compared using identical metrics (AUC, calibration, DCA), the same data split, and 10-fold cross-validation to ensure fairness. SHAP analysis was implemented using Python 3.9.16 with the SHAP library (version 0.41.0). For the random forest (RF) model (a tree-based algorithm), TreeExplainer was used, which is optimized for tree-based models and provides efficient and accurate SHAP value calculation. Other relevant libraries included scikit-learn 1.2.2 (for RF model training), pandas 1.5.3 (data processing), and numpy 1.24.3 (numerical computations).

① Hyperparameter tuning: The RF model was optimized using Grid Search with the following parameter ranges: n_estimators (50–200), max_depth (3–10), and min_samples_split (2–10). The final optimal hyperparameters determined by 10-fold cross-validation were n_estimators = 100, max_depth = 5, and min_samples_split = 5 (seed = 789). The same method was used to optimize models such as XGB and KNN. ② Cross-validation: The training set adopted 10-fold cross-validation to avoid overfitting caused by a single split: the training set was randomly divided into 10 subsets, 9 subsets were used for model training and 1 for testing, and the process was repeated 10 times; the average AUC of the cross-validation was 0.75 (95% CI, 0.70–0.80), and the standard deviation was 0.02, indicating good internal consistency. ③ Benchmark comparison: A performance comparison with ‘prediction using Hunt–Hess score alone’ was added. The results showed that the AUC of prediction using Hunt–Hess score alone was 0.62 (95% CI, 0.55–0.69), which was significantly lower than the 0.76 of the RF model in this study (*p* = 0.02). ④ SHAP sensitivity analysis: A comparison was conducted for missing value imputation methods (multiple imputation vs. single imputation). The results showed that the difference in SHAP mean values of each predictor between the two methods was < 5% (e.g., SHAP mean value of BMI: 0.0706 for multiple imputation vs. 0.0682 for single imputation), indicating that the model is not sensitive to the imputation method.

## 3 Results

### 3.1 Baseline data of training and validation sets and intergroup comparison in the training set

**Figure 1:**
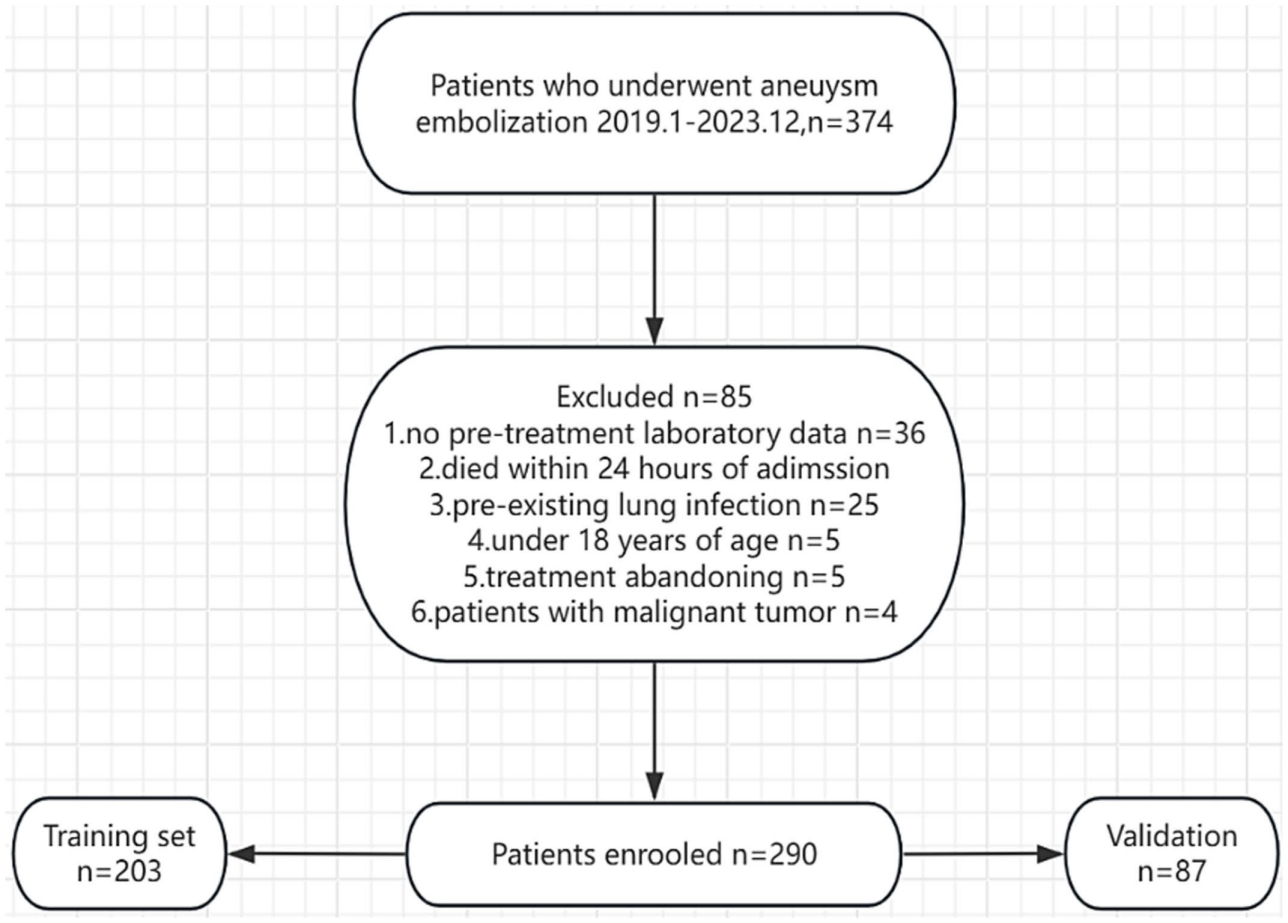
Flow chart of this study.

A total of 375 patients were initially enrolled, with 85 excluded due to exclusion criteria, leaving 290 patients for retrospective analysis (Figure 1). The 85 excluded cases were specifically classified as follows: ① Incomplete medical records (32 cases, 37.6%), mainly due to missing preoperative lactate dehydrogenase (LDH or SIRI test values); ② Complicated with severe cardiovascular diseases (e.g., acute myocardial infarction) or hematological diseases (18 cases, 21.2%); ③ Preoperative community-acquired pneumonia (15 cases, 17.6%); ④ Pseudoaneurysm or non-aneurysmal SAH (12 cases, 14.1%); ⑤ Patients/family members refused treatment (8 cases, 9.4%).

Baseline data of the randomly divided training and validation sets showed no significant statistical differences (*p* > 0.05), indicating comparability (Table 1).

**Table 1:** Comparison for baseline characteristics between test group and train group.

Characteristics	Test (*n* = 87)	Train (*n* = 203)	*p*
Sex			0.128
Female	55 (63.22%)	107 (52.71%)	
Male	32 (36.78%)	96 (47.29%)	
Age	56.00 (50.00,63.00)	55.00 (48.00,64.00)	0.339
Hypertension			0.334
No	33 (37.93%)	72 (35.47%)	
Yes	54 (62.07%)	131 (64.53%)	
Diabetes			1.000
No	81 (93.10%)	188 (92.61%)	
Yes	6 (6.90%)	15 (7.39%)	
GCS score			0.139
≤8	7 (8.05%)	31 (15.27%)	
>8	80 (91.95%)	172 (84.73%)	
Hunt–Hess score			0.162
<3	79 (90.80%)	170 (83.74%)	
≥3	8 (9.20%)	33 (16.26%)	

The training set was grouped by the presence of postoperative pneumonia: the Pneumonia group (75 cases) and the No Pneumonia group (215 cases). Intergroup comparison of all clinical data (with continuous data dichotomized by mean) showed statistical differences in Age, Hunt–Hess score, RBC, Neut, Eo, Plt, Urea, Alb, AGR, AST, lactate dehydrogenase [LDH, D-Dimer, BMI, NLR, SII, PNI, SIRI, and PLR (*p* < 0.05) (Table 2)].

**Table 2:** Comparison of factors between pneumonia group and no pneumonia group.

Characteristics	No pneumonia (*n* = 128)	Pneumonia (*n* = 75)	OR	*p*
Sex				0.778
Female	66 (51.6%)	41 (54.7%)	1	
Male	62 (48.4%)	34 (45.3%)	0.88 (0.50, 1.57)	
Age	52.6 (11.0)	58.5 (11.3)	1.05 (1.02, 1.08)	<0.001
Hypertension				0.785
No	44 (34.4%)	28 (37.3%)	1	
Yes	84 (65.6%)	47 (62.7%)	0.88 (0.49, 1.60)	
Diabetes				0.100
No	122 (95.3%)	66 (88.0%)	1	
Yes	6 (4.69%)	9 (12.0%)	2.74 (0.93, 8.66)	
GCS	1.91 (0.29)	1.75 (0.44)	0.30 (0.14, 0.67)	0.006
Hunt–Hess score				<0.001
<3	117 (91.4%)	53 (70.7%)	1	
≥3	11 (8.59%)	22 (29.3%)	4.35 (2.00, 10.0)	
Hb	130 (18.5)	125 (18.4)	0.99 (0.97, 1.00)	0.055
RBC	4.49 (0.59)	4.27 (0.61)	0.52 (0.31, 0.87)	0.011
HCT	0.38 (0.05)	0.41 (0.43)	1.56 (0.47, 5.21)	0.539
MCV	86.0 (9.25)	86.3 (10.4)	1.00 (0.97, 1.03)	0.819
MCH	29.2 (3.79)	29.2 (4.09)	1.00 (0.93, 1.08)	0.998
MCHC	339 (15.4)	336 (19.2)	0.99 (0.98, 1.01)	0.387
RDW	13.5 (1.98)	15.1 (11.7)	1.05 (0.95, 1.16)	0.270
WBC	13.5 (7.66)	15.0 (5.20)	1.03 (0.99, 1.08)	0.095
Neut	11.0 (5.04)	12.9 (5.02)	1.08 (1.02, 1.14)	0.008
Lymph	1.24 (0.69)	1.17 (0.93)	0.88 (0.60, 1.31)	0.561
Mono	0.62 (0.40)	0.74 (0.50)	1.83 (0.96, 3.50)	0.078
Eo	0.03 (0.08)	0.01 (0.02)	0.00 (0.00, 0.99)	0.010
Baso	0.03 (0.03)	0.03 (0.02)	0.12 (0.00, 17.861)	0.717
Plt	262 (78.8)	287 (74.6)	1.00 (1.00, 1.01)	0.027
Pct	0.25 (0.06)	0.24 (0.06)	0.03 (0.00, 3.32)	0.144
Mpv	10.2 (0.90)	10.3 (0.80)	1.21 (0.87, 1.69)	0.246
Pdw	11.6 (1.92)	11.7 (1.79)	1.03 (0.88, 1.20)	0.701
Pab	0.26 (0.08)	0.25 (0.08)	0.28 (0.01, 13.2)	0.511
Na	139 (4.06)	140 (3.60)	1.03 (0.96, 1.11)	0.352
Cl	103 (4.50)	103 (4.77)	1.02 (0.96, 1.09)	0.464
K	6.55 (30.7)	3.45 (0.53)	0.48 (0.24, 0.97)	0.256
Ca	2.23 (0.14)	2.21 (0.14)	0.38 (0.05, 3.11)	0.363
Urea	4.54 (1.61)	5.19 (1.94)	1.24 (1.05, 1.46)	0.015
Crea	69.3 (27.2)	75.7 (23.4)	1.01 (1.00, 1.02)	0.077
Tp	72.6 (6.40)	72.7 (9.17)	1.00 (0.96, 1.04)	0.947
Alb	42.3 (3.96)	40.0 (7.98)	0.93 (0.88, 0.99)	0.024
AGR	1.44 (0.31)	1.31 (0.28)	0.21 (0.07, 0.65)	0.004
TBIL	12.0 (5.72)	12.0 (5.81)	1.00 (0.95, 1.05)	0.979
DBIL	4.79 (5.66)	4.69 (2.91)	1.00 (0.94, 1.06)	0.879
ALT	20.6 (13.6)	20.7 (11.7)	1.00 (0.98, 1.02)	0.954
AST	22.6 (13.2)	29.1 (17.0)	1.03 (1.01, 1.05)	0.005
ALP	71.1 (20.1)	70.3 (20.9)	1.00 (0.98, 1.01)	0.798
GCT	32.1 (28.5)	44.7 (114)	1.00 (1.00, 1.01)	0.355
LDH	196 (42.6)	221 (51.2)	1.01 (1.00, 1.02)	0.001
ADA	9.69 (4.45)	10.9 (4.40)	1.06 (1.00, 1.13)	0.057
FIB	3.34 (0.72)	3.63 (1.18)	1.41 (1.02, 1.94)	0.057
D-Dimer	2039 (2889)	3,644 (4877)	1.00 (1.00, 1.00)	0.011
MI	23.6 (2.98)	22.4 (3.39)	0.88 (0.80, 0.97)	0.012
SBP	146 (24.1)	146 (29.3)	1.00 (0.99, 1.01)	0.999
DBP	87.2 (14.7)	84.1 (17.2)	0.99 (0.97, 1.01)	0.186
NLR	11.8 (8.49)	14.3 (8.69)	1.03 (1.00, 1.07)	0.048
SII	3,079 (2446)	4,130 (2681)	1.00 (1.00, 1.00)	0.006
PNI	48.5 (5.06)	45.9 (9.55)	0.94 (0.90, 0.99)	0.030
SIRI	7.15 (8.01)	10.5 (9.67)	1.04 (1.01, 1.08)	0.012
PLR	262 (139)	315 (168)	1.00 (1.00, 1.00)	0.023

### 3.2 Multi-step feature selection

Univariate logistic regression, LASSO regression, and Boruta algorithm were used for multi-step feature selection.

*Univariate logistic regression*: PLR, PNI, SIRI, SII, NLR, BMI, Hunt–Hess score, GCS score, D-Dimer, FIB, lactate dehydrogenase [LDH, AST, AGR, Alb, Urea, K, Plt, Eo, Neut, RBC, and Age were identified as significant predictors (*p* < 0.05) (Table 3)].

**Table 3:** Univariate logistic regression analysis of clinical factors in training group.

Characteristics	B	SE	OR	CI	*Z*	*p*	
Age	0.049	0.01414	1.051	1.051 (1.023–1.081)	3.488	<0.001	
SEX	−0.125	0.29169	0.883	0.883 (0.497–1.563)	−0.427	0.669	
Hunt–Hess score	1.485	0.4047	4.415	4.415 (2.036–10.08)	3.669	<0.001	
GCS score	−1.188	0.40304	0.305	0.305 (0.135–0.664)	−2.947	<0.01	
Diabetes	1.02	0.54875	2.773	2.773 (0.958–8.59)	1.858	0.063	
Hypertension	−0.129	0.30269	0.879	0.879 (0.487–1.599)	−0.425	0.671	
PLR	0.002	0.00096	1.002	1.002 (1–1.004)	2.358	<0.05	
SIRI	0.044	0.01743	1.045	1.045 (1.011–1.083)	2.516	<0.05	
PNI	−0.057	0.02418	0.944	0.944 (0.897–0.987)	−2.375	<0.05	
SII	0	0.00006	1	1 (1–1)	2.719	<0.01	
NLR	0.033	0.01691	1.034	1.034 (1–1.069)	1.964	<0.05	
DBP	−0.013	0.00945	0.987	0.987 (0.969–1.005)	−1.378	0.168	
SBP	0	0.00559	1	1 (0.989–1.011)	−0.001	0.999	
MI	−0.126	0.04904	0.882	0.882 (0.799–0.969)	−2.564	<0.05	
D-Dimer	0	0.00004	1	1 (1–1)	2.601	<0.01	
FIB	0.342	0.16445	1.407	1.407 (1.03–1.976)	2.077	<0.05	
ADA	0.062	0.03298	1.064	1.064 (0.998–1.137)	1.879	0.060	
LDH	0.011	0.0034	1.011	1.011 (1.005–1.019)	3.339	<0.001	
GCT	0.003	0.00277	1.003	1.003 (0.998–1.011)	0.965	0.335	
ALP	−0.002	0.00719	0.998	0.998 (0.984–1.012)	−0.26	0.795	
AST	0.03	0.01073	1.031	1.031 (1.01–1.054)	2.812	<0.01	
ALT	0.001	0.01127	1.001	1.001 (0.978–1.023)	0.056	0.956	
DBIL	−0.004	0.03116	0.996	0.996 (0.918–1.059)	−0.131	0.896	
TBIL	−0.001	0.02544	0.999	0.999 (0.948–1.05)	−0.027	0.978	
AGR	−1.54	0.56738	0.214	0.214 (0.067–0.619)	−2.715	<0.01	
Alb	−0.072	0.02932	0.931	0.931 (0.875–0.982)	−2.441	<0.05	
Tp	0.001	0.0194	1.001	1.001 (0.964–1.041)	0.074	0.941	
Crea	0.01	0.00581	1.01	1.01 (0.999–1.022)	1.645	0.100	
Urea	0.211	0.08524	1.235	1.235 (1.049–1.468)	2.477	<0.05	
Ca	−0.973	1.07573	0.378	0.378 (0.041–2.878)	−0.904	0.366	
K	−0.733	0.35606	0.48	0.48 (0.23–0.953)	−2.059	<0.05	
Cl	0.024	0.03172	1.024	1.024 (0.962–1.09)	0.746	0.456	
Na	0.034	0.03771	1.035	1.035 (0.961–1.116)	0.904	0.366	
Pab	−1.285	1.9718	0.277	0.277 (0.005–11.74)	−0.652	0.515	
Pdw	0.029	0.07777	1.03	1.03 (0.883–1.2)	0.379	0.705	
Mpv	0.191	0.1697	1.211	1.211 (0.869–1.696)	1.126	0.260	
Pct	−3.5	2.39771	0.03	0.03 (0–2.923)	−1.46	0.144	
Plt	0.004	0.00193	1.004	1.004 (1–1.008)	2.14	<0.05	
Baso	−2.113	6.07325	0.121	0.121 (0–13,294)	−0.348	0.728	
Eo	−9.03	4.60336	0	0 (0–0.206)	−1.962	<0.05	
Mono	0.607	0.32976	1.835	1.835 (0.967–3.559)	1.841	0.066	
Lymph	−0.126	0.20065	0.882	0.882 (0.57–1.276)	−0.627	0.531	
Neut	0.076	0.02963	1.079	1.079 (1.02–1.146)	2.575	<0.05	
WBC	0.033	0.0233	1.034	1.034 (0.991–1.087)	1.419	0.156	
RDW	0.048	0.0529	1.049	1.049 (0.991–1.195)	0.908	0.364	
MCHC	−0.008	0.00857	0.992	0.992 (0.975–1.009)	−0.914	0.360	
MCH	0	0.03742	1	1 (0.93–1.078)	0.003	0.997	
MCV	0.004	0.01517	1.004	1.004 (0.975–1.035)	0.237	0.813	
HCT	0.446	0.61439	1.561	1.561 (0.491–12.81)	0.725	0.468	
RBC	−0.657	0.26352	0.519	0.519 (0.303–0.855)	−2.492	<0.05	
Hb	−0.015	0.00793	0.985	0.985 (0.97–1)	−1.905	0.057	
MCV	0.004	0.01517	1.004	1.004 (0.975–1.035)	0.237	0.813	0.813
HCT	0.446	0.61439	1.561	1.561 (0.491–12.81)	0.725	0.468	0.468
RBC	−0.657	0.26352	0.519	0.519 (0.303–0.855)	−2.492	<0.05	0.013
Hb	−0.015	0.00793	0.985	0.985 (0.97–1)	−1.905	0.057	0.057
Age	0.049	0.01414	1.051	1.051 (1.023–1.081)	3.488	<0.001	0.000
SEX2	−0.125	0.29169	0.883	0.883 (0.497–1.563)	−0.427	0.669	0.669

**Figure 2:**
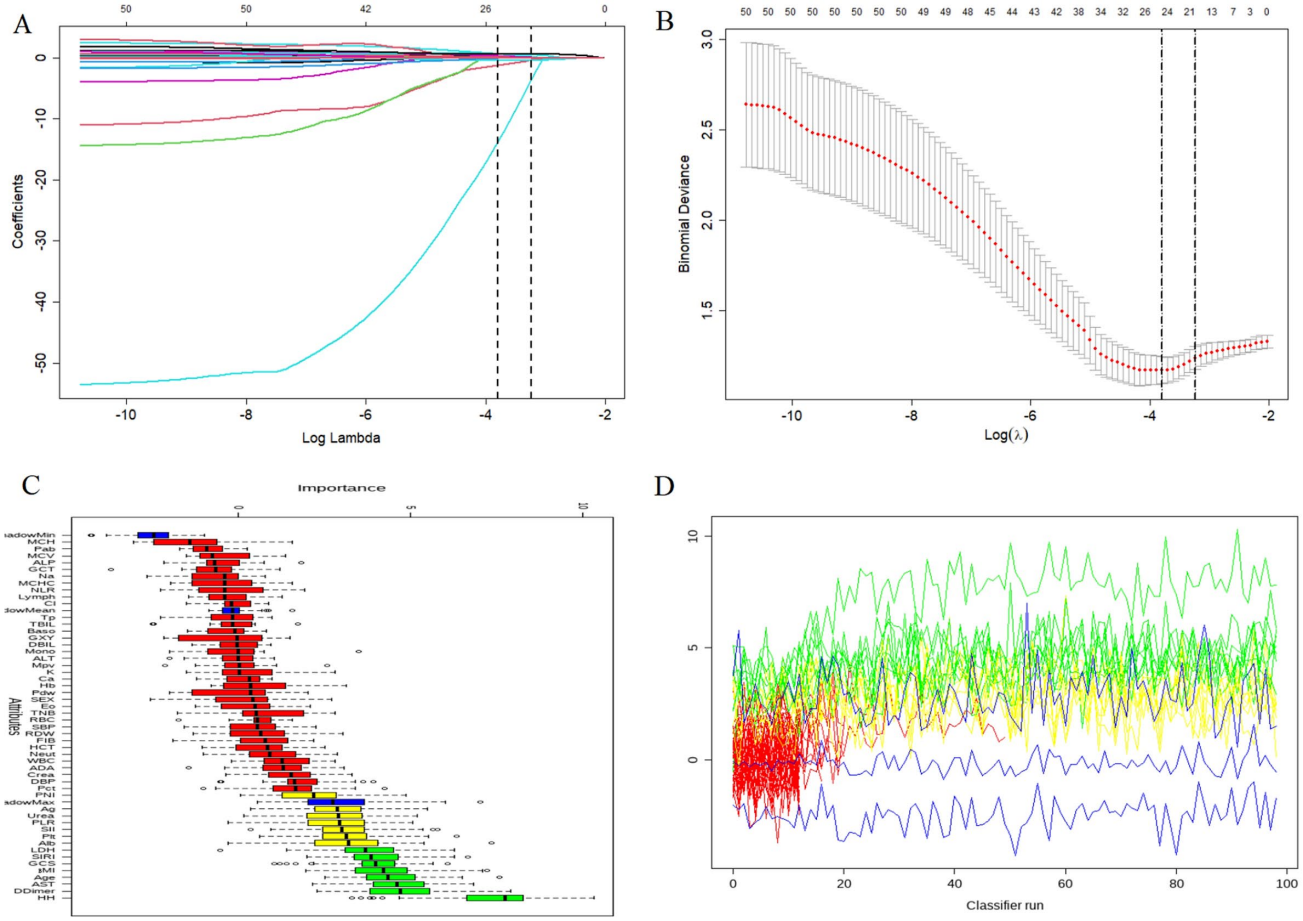
Filter single factor. **(A,B)** LASSO regression analysis; **(C,D)** Boruta feature selection.

*LASSO regression*: With *λ* set to Lambda.min for model accuracy, Age, RBC, HCT, RDW, Mono, Eo, Plt, Pct, Mpv, Urea, Alb, AGR, AST, lactate dehydrogenase [LDH, FIB, D-Dimer, Diabetes, Hunt–Hess score, BMI, SII, and SIRI were screened as risk factors (Figures 2A,B)].

*Boruta algorithm*: Recommended retained factors were lactate dehydrogenase [LDH, SIRI, GCS score, BMI, Age, AST, D-Dimer, and Hunt–Hess score (Figures 2C,D)].

The common factors across the three methods were lactate dehydrogenase (LDH, SIRI, BMI, Age, AST, D-Dimer, and Hunt–Hess score).

### 3.3 Multivariate logistic regression, nomogram construction, and validation

Based on the screening results [lactate dehydrogenase (LDH, SIRI, BMI, Age, AST, D-Dimer, Hunt–Hess score)], multivariate analysis was performed (Table 4), identifying independent predictors of postoperative pneumonia as lactate dehydrogenase (LDH (OR[95% CI] = 1.008 [1.001–1.015], *p* < 0.05), SIRI (OR[95% CI] = 1.041 [1.003–1.082], *p* < 0.05), HH (OR[95% CI] = 3.117 [1.336–7.566], *p* < 0.01), and Age (OR[95% CI] = 1.045 [1.015–1.078], *p* < 0.01). In this study, 7 features [lactate dehydrogenase (LDH, SIRI, BMI, Age, AST, D-Dimer, Hunt–Hess score)] were included in the ML model instead of only retaining the 4 significant features [lactate dehydrogenase (LDH, SIRI, Hunt–Hess score, Age)] from multivariate regression. The reasons are as follows: ① ML models (e.g., RF) can capture the interaction between features—for example, the combined effect of BMI and AST (the stroke-associated pneumonia (SAP) risk of patients with high BMI and AST > 40 U/L was 2.8 times that of patients with low BMI and normal AST), while traditional multivariate regression tends to ignore such non-linear associations. ② Some features that are “not significant in univariate analysis” (e.g., D-Dimer) still have predictive value in multi-feature combinations. SHAP analysis showed that when D-Dimer > 1.5 mg/L, the predicted probability could increase by 8%–12%. ③ Including multi-dimensional features can improve the adaptability of the model to different clinical scenarios.

**Table 4:** Multivariate logistic regression analysis of clinical factors in training group.

Characteristics	B	SE	OR	CI	*Z*	*p*
Age	0.045	0.01544	1.046	1.045 (1.015–1.078)	2.888	<0.01
LDH	0.008	0.00357	1.008	1.008 (1.001–1.015)	2.272	<0.05
SIRI	0.04	0.01922	1.041	1.041 (1.003–1.082)	2.091	<0.05
MI	−0.077	0.05395	0.926	0.925 (0.830–1.027)	−1.427	0.153
HH2	1.137	0.43883	3.118	3.117 (1.336–7.566)	2.591	<0.01

Stratified analysis showed that the combined OR of high BMI (>28 kg/m^2^) and elevated AST (>40 U/L) for stroke-associated pneumonia (SAP) was 2.8 (95% CI, 1.53–5.12, *p* < 0.001); the incremental AUC of adding D-Dimer to the 4 significant independent predictors was 0.08 (95% CI, 0.03–0.13), indicating its supplementary predictive value.

The RF model was tested for overfitting via out-of-bag (OOB) error (OOB error = 0.21) and 1,000 times Bootstrap resampling (AUC fluctuation range 0.72–0.78, no significant drift), confirming no overfitting caused by retaining seven features.

**Figure 3:**
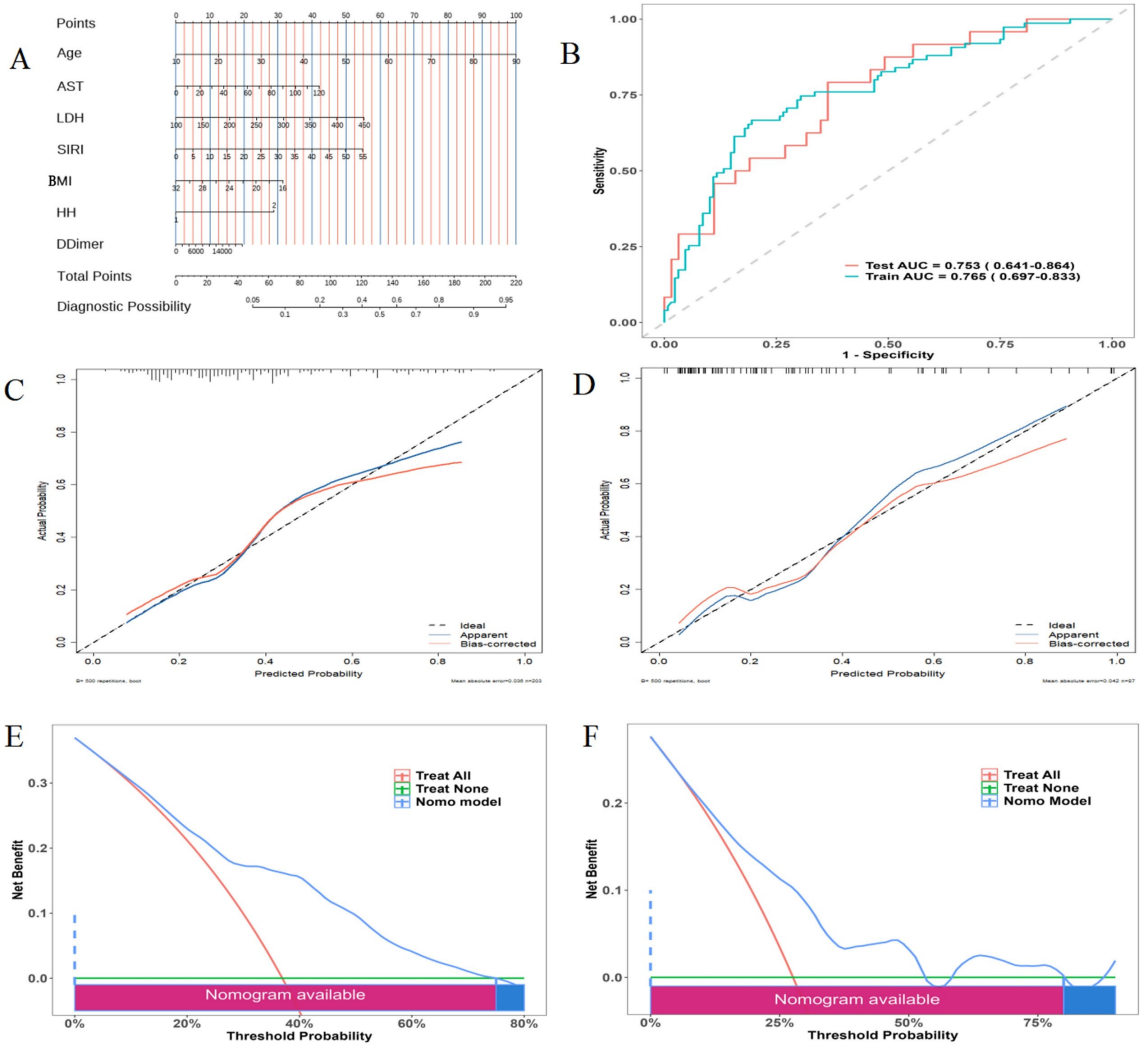
Establishment and verification of nomogram. **(A)** Nomogram; **(B)** ROC curves for the training and validation sets; **(C,D)** Calibration curves for training and validation sets; **(E,F)** DCA curves for training and validation sets.

A nomogram model was constructed using R studio and JD_DCPM software (Figure 3A). The training set ROC curve showed an AUC of 0.765, and the validation set AUC was 0.753, both indicating good predictive efficacy (Figure 3B). Calibration curves for the training and validation sets showed minimal mean errors, with actual and theoretical predictions nearly overlapping (Figures 3C,D). DCA curves showed that when the clinical decision threshold probability was in the range of 10–80%, the nomogram model had a higher clinical net benefit than the simple clinical judgment; when the threshold probability was <10% or >80%, the net benefit of the model was limited, indicating the specific clinical application scope of the model (Figures 3E,F).

Results of target trial emulation showed that after PSM matching, the standardized mean difference (SMD) of all confounding factors (Age, BMI, Hunt–Hess score, etc.) was <0.1, and the variance ratio (VR) was between 0.8 and 1.2, indicating that the two groups were well balanced; the common support diagram showed that 95% of the samples in the exposed and control groups fell within the common support range, and no sample was outside the range, ensuring the validity of matching. The incidence of stroke-associated pneumonia (SAP) in the exposed group (SIRI>1.5, *n* = 92) was 38.0% (35/92), which was significantly higher than that in the control group (SIRI ≤ 1.5, *n* = 92) at 18.5% (17/92), with RR = 2.05 (95% CI: 1.23–3.42, *p* = 0.006). *E*-value analysis was used to evaluate the impact of unmeasured confounding on the association between SIRI and stroke-associated pneumonia (SAP). The E-value of the risk ratio (RR = 2.05) was 2.31, indicating that an unmeasured confounding factor needs to have an RR of 2.31 with both SIRI and stroke-associated pneumonia (SAP) to completely offset the observed association, suggesting that the study results are robust to unmeasured confounding. Similarly, the risk of stroke-associated pneumonia (SAP) in patients with Hunt–Hess grade IV-V was significantly higher than that in patients with grade I–III (RR = 3.12, 95% CI: 1.68–5.79, *p* < 0.001), further supporting the clinical significance of key predictors. This supports an association between elevated SIRI and increased stroke-associated pneumonia (SAP) risk, with residual confounding cannot be completely excluded. Similarly, the risk of stroke-associated pneumonia (SAP) in patients with Hunt–Hess grade IV–V was significantly higher than that in patients with grade I–III (RR = 3.12, 95% CI: 1.68–5.79, *p* < 0.001), further supporting the clinical significance of key predictors.

### 3.4 Machine learning performance comparison and SHAP interpretation

The RF model showed the most stable performance in both training and validation sets, and was identified as the primary analytic model; the nomogram, as a supplementary tool, has good clinical applicability due to its visuality and simplicity.

**Figure 4:**
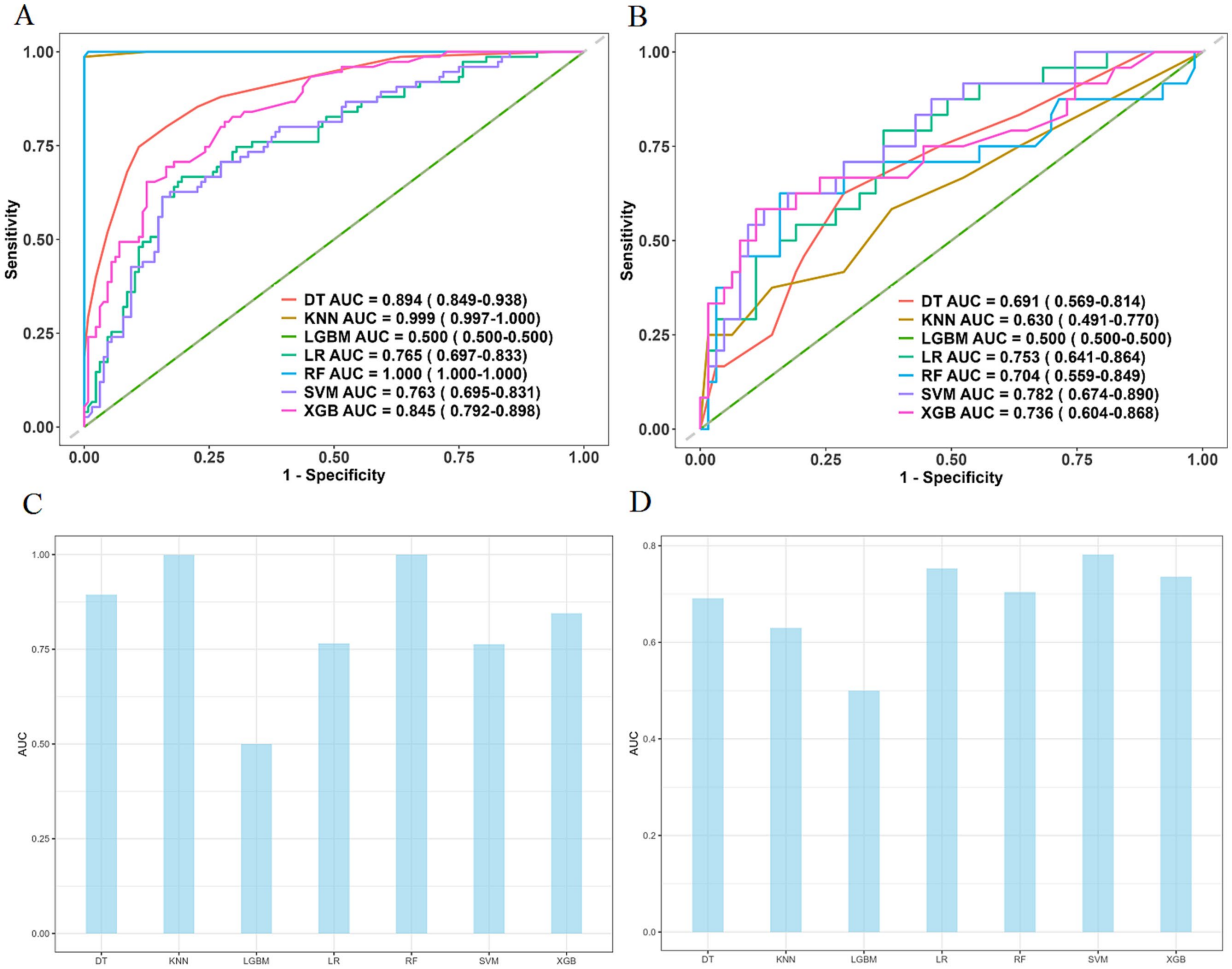
Comparison of seven machine learning models. **(A)** and **(B)** show ROC curves of the seven machine learning models; **(C)** and **(D)** show bar charts of AUC values.

Performance comparisons of seven machine learning algorithms are shown in Table 5, with ROC curves and bar charts visually displaying model performance (Figure 4). The RF model showed stable performance and good efficacy in both training and validation sets. Based on the RF model, SHAP analysis was conducted to evaluate risk factor importance and individual impacts. The discrepancy between SHAP and logistic regression results reflects different aims: SHAP quantifies predictive contribution (including non-linear interactions, e.g., BMI + AST), while logistic regression assesses independent inferential association. RF captures that high BMI combined with AST > 40 U/L increases stroke-associated pneumonia (SAP) risk 2.8-fold, a relationship ignored by linear logistic regression.

**Table 5:** Comparison of AUC for various machine learning models.

Models	Training set AUC	Validation set AUC
DT	0.894 (0.849–0.938)	0.691 (0.569–0.814)
KNN	0.999 (0.997–1.000)	0.630 (0.491–0.770)
LGBM	0.500 (0.500–0.500)	0.500 (0.500–0.500)
LR	0.765 (0.697–0.833)	0.753 (0.641–0.864)
RF	1.000 (1.000–1.000)	0.704 (0.559–0.849)
SVM	0.763 (0.695–0.831)	0.782 (0.674–0.890)
XGB	0.845 (0.792–0.898)	0.736 (0.604–0.868)

**Figure 5:**
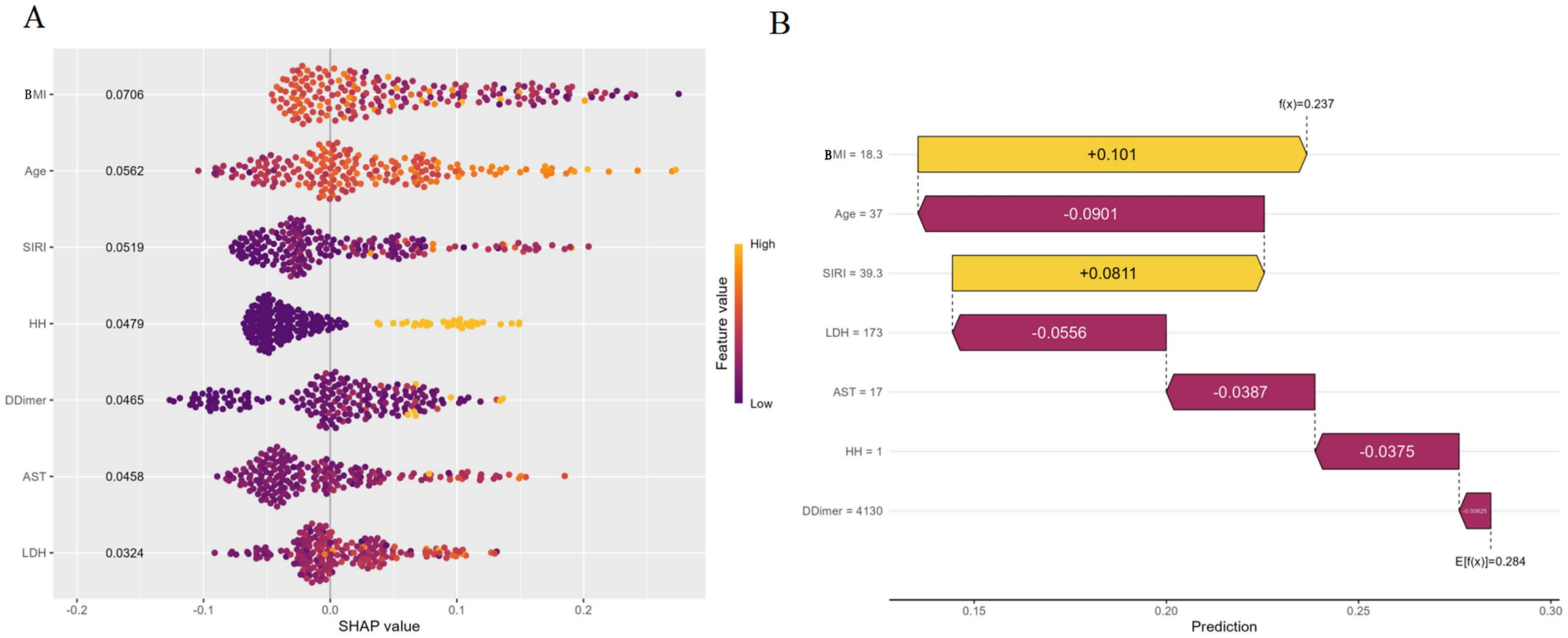
SHAP interpretation and importance ranking of predictive factors for RF machine learning models. **(A)** The SHAP Bee Swarm Plot; **(B)** The SHAP waterfall plot.

SHAP bee swarm plot (Figure 5A): The mean SHAP values for BMI, Age, SIRI, Hunt–Hess score, D-Dimer, AST, and lactate dehydrogenase (LDH were 0.0706, 0.0562, 0.0519, 0.0479, 0.0465, 0.0458, and 0.0324, respectively). BMI showed the largest SHAP value range, indicating the most significant impact on model predictions, followed by Age, SIRI, HH, D-Dimer, AST, and lactate dehydrogenase (LDH.

Waterfall plot for a randomly sampled individual (Figure 5B): The plot showed BMI had the longest right-pointing arrow, indicating the greatest positive impact on predictions (higher BMI increased pneumonia probability). Age had the second-longest left-pointing arrow, indicating a negative impact (younger age reduced pneumonia probability). The sum of all features’ SHAP values gave a predicted probability of 0.237.

## 4 Discussion

This study constructed a SHAP-explainable random forest model to predict risk factors for stroke-associated pneumonia [stroke-associated pneumonia (SAP)] in patients with aneurysmal subarachnoid hemorrhage (aSAH). This study’s primary model is the RF random forest model combined with SHAP interpretation, which balances predictive performance and interpretability. The following discussion focuses on key findings from this primary model, with comparisons to the nomogram where relevant.

As an indicator of nutritional and metabolic status, BMI plays a key role in predicting inflammation-related complications. SHAP analysis in the RF model showed that BMI contributed the most to predictions, highlighting the critical role of nutritional management in reducing pneumonia risk (24, 25). Age, a common demographic factor, has been proven in multiple studies to be significantly positively correlated with stroke-associated pneumonia (SAP) risk. In RF model feature analysis, Age was frequently identified as a top feature by SHAP, with high values (e.g., ≥60 years) associated with increased pneumonia incidence, reflecting immune decline and vulnerability in elderly patients (26, 27).

Furthermore, the management of stroke-associated pneumonia (SAP) in aSAH patients should be integrated with interventions for neurobehavioral deficits. Recent studies have shown that the persistent inflammatory state caused by stroke-associated pneumonia (SAP) may exacerbate neurocognitive impairments (such as inattention and decreased executive function) after brain injury. However, targeted infection control (e.g., early antibiotic intervention, respiratory support) can indirectly improve the long-term neurobehavioral prognosis of patients by reducing the inflammatory burden (28). If the prediction model constructed in this study is applied in clinical practice, it can provide a management window for synchronously optimizing neurological recovery by early identifying high-risk patients with stroke-associated pneumonia (SAP) and initiating interventions. For example, in patients with Hunt–Hess grade IV-V and elevated SIRI, combining neurorehabilitation training while preventing pneumonia may further reduce the disability rate.

This study must acknowledge the significant heterogeneity of aSAH patients in the ICU—for example, the difference in stroke-associated pneumonia (SAP) risk between ventilated patients (42 cases, 14.5% in this study) and non-ventilated patients (incidence of 47.6% in ventilated patients vs. 19.8% in non-ventilated patients, *p* < 0.001), and the impact of different surgical timings (within 24 h vs. 24–72 h after onset) on predictors (e.g., SIRI has a higher predictive weight in patients with early surgery). Studies have confirmed that ignoring patient heterogeneity may lead to decreased model performance in specific subgroups (29). Based on the data of this study, we found that: ① In the subgroup of Age ≥ 60 years, the SHAP contribution value of BMI (0.091) was significantly higher than that in the young subgroup (0.052); ② In the subgroup of Hunt–Hess grade I–II, the predictive value of D-Dimer was more prominent (AUC increased by 0.08). In the future, subgroup-specific model construction will be carried out through multicenter data to further optimize individualized intervention plans.

The association between SIRI and aSAH-related pneumonia has been validated in clinical studies. High SIRI values reflect an inflammatory state of neutrophilia and lymphopenia, increasing pneumonia risk, and SHAP analysis emphasized its significant contribution to the prediction model (30, 31). The Hunt–Hess score, used to assess the initial neurological severity of aSAH, was an independent predictor of stroke-associated pneumonia (SAP) in this study. Literature supports its role in predicting pneumonia, as high scores (IV–V) are often associated with postoperative complications like stroke-associated pneumonia (SAP) due to severe neurological injury leading to dysphagia and increased lung infection risk, frequently ranking among top features in model feature (32–34).

High D-Dimer levels are associated with inflammatory activation and thrombotic risk in aSAH patients, directly linking to the pathogenesis of lung infections (e.g., microthrombus-induced hypoxia), and were identified as top features in SHAP interpretation of the RF model (26, 33). AST levels were confirmed by SHAP as significant factors in predicting postoperative inflammatory responses, reflecting the role of tissue damage-released enzymes in stroke-associated pneumonia (SAP) pathogenesis (35). It is worth noting that ferritin toxicity is a potential mechanism contributing to stroke-associated pneumonia (SAP) susceptibility in aSAH patients – a hypothesis that requires validation in prospective studies. Studies have confirmed that free iron released from red blood cell rupture after aSAH can induce overexpression of ferritin. Elevated ferritin levels significantly increase the risk of pulmonary infection by promoting oxidative stress and inhibiting macrophage phagocytosis (36). Although ferritin was not directly included as an indicator in this study, the observed elevation of lactate dehydrogenase [LDH (reflecting cell damage) and SIRI (reflecting inflammatory status)] may be indirectly associated with the ferritin toxicity pathway. Future studies could incorporate ferritin into the prediction model to further enhance the explanatory power for infection mechanisms. Lactate dehydrogenase LDH is more directly associated with cellular energy metabolism and hypoxic injury (37). BMI reflects metabolic/nutritional status, AST reflects tissue damage, and D-Dimer reflects coagulation/inflammatory activation—these three factors together with the four independent predictors [Age, SIRI, Hunt–Hess score, lactate dehydrogenase (LDH)] construct a multi-dimensional risk assessment system for stroke-associated pneumonia (SAP) from metabolic, inflammatory, coagulation, and neurological severity perspectives, and none of them are redundant noise variables. The comprehensive application of these factors provides a multidimensional risk assessment framework for individualized management of aSAH patients.

In previous literature, SHAP has been widely used to explain the prediction mechanisms of RF models, visually displaying the contribution of each feature (e.g., BMI, Age) to individual predictions. This not only provides transparency to black-box models but also helps clinicians identify key intervention points (38). Compared with other methods, the SHAP-combined RF model outperforms traditional scoring systems (e.g., relying solely on linear regression) in interpretability, supporting its potential as a clinical decision-making aid (39).

Regarding key issues in clinical practice for stroke-associated pneumonia (SAP) prevention: ① For vaccination upon admission, this study did not collect data on influenza vaccine or vaccination history, so it was unable to evaluate their protective effect against stroke-associated pneumonia (SAP). However, existing evidence shows that preoperative vaccination with pneumococcal vaccine in elderly or immunocompromised patients can reduce the rate of postoperative pulmonary infection (40). This indicator could be included in future studies to improve the model. ② For infection risk mitigation measures, based on the predictors identified in this study, targeted interventions are recommended for high-risk patients (e.g., Age ≥ 60 years, BMI > 28 kg/m^2^, SIRI > 1.5), including enhanced oral care, elevating the head of the bed by 30°, early enteral nutrition support, and avoiding unnecessary invasive procedures. ③ For genetic factors, this study adopted a single-center retrospective design and did not involve genetic polymorphism data (e.g., the inflammation-related gene IL-6 rs1800795). However, genetic variations may affect infection susceptibility by regulating the immune response (41), which will be a key direction for subsequent multicenter studies.

However, this study has limitations: (1) This study is a single-center retrospective analysis, with all data sourced from Huizhou Central People’s Hospital. There may be biases related to regional demographic characteristics (e.g., Han population in southern China) and treatment protocols (e.g., post-embolization monitoring procedures), which limit the external validity of the model. For instance, the predictive performance of the model may decrease in centers in northern China or those using different surgical methods (e.g., surgical clipping). To address this issue, we plan to conduct multicenter external validation across 3 tertiary hospitals in Guangdong Province (Gaozhou People’s Hospital, The First Affiliated Hospital of Shaoyang University, and an additional center) between 2025 and 2026. The target sample size is 500 aSAH patients, with primary evaluation metrics including discrimination (AUC), calibration (Hosmer–Lemeshow test), and reclassification (net reclassification improvement, NRI). (2) The biological mechanisms of some factors [e.g., lactate dehydrogenase (LDH and AST)] require validation in large prospective studies; (3) This study did not include a comparative analysis of ventilator management differences between centers. However, variations in ventilation mode selection and weaning timing judgment among different centers may affect the incidence of stroke-associated pneumonia (SAP). Future multicenter studies should collect details of ventilator management and evaluate its impact on the predictive performance of the model through subgroup analysis to improve the applicability of the model in different clinical scenarios. Future work should integrate multicenter data to optimize model robustness and develop web-based tools (e.g., RF model deployment) to enhance clinical accessibility.

## 5 Conclusion

This study identifies BMI, Age, SIRI, Hunt–Hess score, D-Dimer, AST, and lactate dehydrogenase LDH as key predictors of postoperative stroke-associated pneumonia (SAP) in aSAH, and demonstrates the efficient performance of the RF random forest model (combined with SHAP interpretation) as the primary analytic model; the nomogram as a supplementary tool provides a convenient visual method for clinical rapid risk assessment. Combined with SHAP’s interpretable framework, this model not only improves prediction accuracy, but also may aid in stroke-associated pneumonia (SAP) risk stratification and inform personalized healthcare strategies. These results emphasize the value of integrating inflammatory, neurological, and metabolic indicators in preventing stroke-associated pneumonia (SAP), and may provide a foundation for shifting clinical practice toward preventive management, with specific action thresholds requiring further prospective validation. The model is intended for use within 24 h after aSAH admission to identify high-risk patients early and guide timely interventions. This study lacks external validation, which limits the generalizability of the model. Future multicenter studies are needed to confirm its performance in diverse clinical settings.

## Data Availability

The raw data supporting the conclusions of this article will be made available by the authors, without undue reservation.

## Footnotes

1
https://doi.org/10.1016/j.lers.2025.01.001

## Glossary

Hb: Hemoglobin
RBC: Red blood cell count
HCT: Hematocrit
MCV: Mean corpuscular volume
MCH: Mean corpuscular hemoglobin
MCHC: Mean corpuscular hemoglobin concentration
RDW: red cell distribution width
WBC: White blood cell count
Neut: Neutneutrophils
Lymph: Lymphocytes
Mono: Monocytes
Eo: Eosinophils
Baso: Basophils
Plt: Platelet count
Pct: Plateletcrit
Mpv: Mean platelet volume
Pdw: Platelet distribution width
Pab: Prealbumin
Na: Sodium
Cl: Chloride
K: Potassium
Ca: Calcium
Crea: Creatinine
Tp: Total protein
Alb: Albumin
AGR: Albumin/globulin ratio
TBIL: Total bilirubin
DBIL: Direct bilirubin
ALT: Alanine aminotransferase
AST: Aspartate aminotransferase
ALP: Alkaline phosphatase
GGT: Gamma-glutamyl transferase
LDH: Lactate dehydrogenase
ADA: Adenosine deaminase
FIB: Fibrinogen
SBP: Systolic blood pressure
DBP: Diastolic blood pressure
NLR: Neutrophil-to-lymphocyte ratio
SII: Systemic immune-inflammation index
PNI: Prognostic nutritional index
SIRI: Systemic inflammation response index
PLR: Platelet-to-lymphocyte ratio
OR: Odds ratio
CI: Confidence interval
AUC: The area under the curve
DCA: Decision curve analysis
ROC: Receiver operating characteristic
